# Cold Plasma Technology Based Eco-Friendly Food Packaging Biomaterials

**DOI:** 10.3390/polym16020230

**Published:** 2024-01-14

**Authors:** Chandrima Karthik, Rubie Mavelil-Sam, Sabu Thomas, Vinoy Thomas

**Affiliations:** 1 Department of Mechanical and Materials Engineering, University of Alabama at Birmingham (UAB), Birmingham, AL 35294, USA; ckarthik@uab.edu; 2 College of Science and Engineering, James Cook University, Townsville, QLD 4811, Australia; rubie.mavelilsam@my.jcu.edu.au; 3 School of Nanoscience and Nanotechnology, Mahatma Gandhi University, Kottayam 686560, India; sabuthomas@mgu.ac.in; 4 Trivandrum Engineering Science and Technology Research Park (TrEST), Thiruvananthapuram 695016, India

**Keywords:** cold plasma technology, green packaging, biomaterials

## Abstract

Biopolymers have intrinsic drawbacks compared to traditional plastics, such as hydrophilicity, poor thermo-mechanical behaviours, and barrier characteristics. Therefore, biopolymers or their film modifications offer a chance to create packaging materials with specified properties. Cold atmospheric plasma (CAP) or Low temperature plasma (LTP) has a wide range of applications and has recently been used in the food industry as a potent tool for non-thermal food processing. Though its original purpose was to boost polymer surface energy for better adherence and printability, it has since become an effective technique for surface decontamination of food items and food packaging materials. These revolutionary innovative food processing methods enable the balance between the economic constraints and higher quality while ensuring food stability and minimal processing. For CAP to be considered as a viable alternative food processing technology, it must positively affect food quality. Food products may have their desired functional qualities by adjusting the conditions for cold plasma formation. Cold plasma is a non-thermal method that has little effects on the treated materials and is safe for the environment. In this review, we focus on recent cold plasma advances on various food matrices derived from plants and animals with the aim of highlighting potential applications, ongoing research, and market trends.

## 1. Introduction

Plasma, the fourth state of matter, consist of ions, electrons, and neutral molecules. In some plasmas, the density of charged particles is substantially lower than that of neutral species in because of their low level of ionization. As a result, electrons have kinetic energy that is substantially higher than that of bulk neutrals due to the interaction with the applied field, leading to non-equilibrium or cold atmospheric plasma. It is also known as non-thermal plasma because non-equilibrium plasma does not increase the temperature of the gas [1]. Plasma comes from a variety of sources, depending on the electrode and energy source used. Electron energy, density, and breakdown voltage are used to qualify and quantify plasma generators. Atmospheric pressure or low pressure both produce non-thermal plasmas. Vacuum maintenance for low-pressure plasma discharges necessitates expensive process chambers. The advantages of cold plasmas produced at atmospheric pressure outweigh the need for such arrangements [2]. Natural polymers like polysaccharides, proteins, and lipids are now frequently used in the production of biodegradable packaging materials for packaging due to rising consumer demand for products that are biodegradable, environmentally friendly, long-lasting, and safe [3,4]. The safety issues related to packaged foods are reduced by using various physical and chemical treatment techniques. As an illustration, the food business frequently uses preservatives like sorbic and benzoic acids. But certain chemical substances can also result in issues with environmental safety and have mutagenic and carcinogenic properties. But, used of cold atmospheric plasma can resolve these existing problems. By removing heat from the processing process, these novel non-thermal technologies improve sensory qualities and nutrition retention while preventing the growth of microorganisms. By changing the cell membrane or eliminating the genetic material of bacteria, non-thermal processing sterilizes food and the packaging.

**Figure 1:**
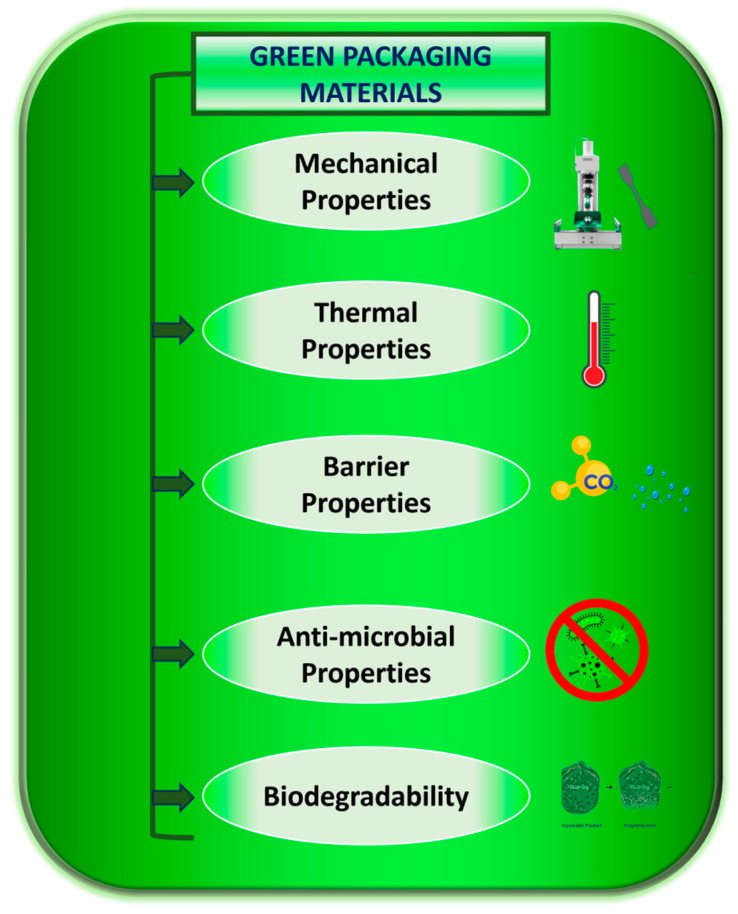
Pillar properties for a good green packaging material.

Polymers are widely used in a variety of industries, including biomedicine, manufacturing, and agriculture due to their strong chemical resistance, flexibility, and low density [5]. Some polymers have boundaries, which make them inappropriate for some applications. These downsides include poor adhesion, wettability, and low surface free energy, all of which are brought on by low surface polar groups [6]. The most sustainable method of processing is plasma. Since it only modifies the surface properties of the polymer without influencing the bulk properties, it is a low-temperature, low-cost, non-toxic, and efficient method of surface modification [7]. Figure 1 shows the various property requirements for polymeric packaging materials.

## 2. Cold Plasma Technology for Property Enhancements in Sustainable Packaging

Numerous variables, such as the feeding gas composition, input power, moisture, applied voltage, etc., affect plasma characteristics. Corona discharge plasma, microwave plasma, inductively coupled plasma, and dielectric barrier discharge (DBD), radio frequency discharge plasma (RF Plasma) are the primary sources of LTP [8]. In addition, the corona discharge process is considered as one of the most important type in this food packaging sector for functionalizing or surface-printing packaging materials [9]. Table 1 shows various sources of low temperature plasma systems used in the processing of polymeric packaging materials. 

The utilization of plasma in the food industry is still in its early phases. Cold plasma has demonstrated effectiveness in the treatment of biofilms as well as in the decontamination of food items such fruits, vegetables, poultry, meat, cereals, spices, etc. [20]. Gas composition, gas pressure, gas flow rate, power, packaging etc can alter the sterilization power of plasma. A significant factor that influences how effective plasma is the gas’s composition. The type of active species that are present will change depend on the input gas [21]. According to Edelblute et al., tripling the gas flow increased the number of spores destroyed in 5 min by three orders of magnitude [22]. With increasing pressure and rising gas temperature, the plasma volume may decrease. At pressure = 400 mtorr, Escherichia coli bacteria were seen to be destroyed in the work published by Mok and Song, whereas at *p* = 43 and 220 mtorr, seen to be completely inactivated [23]. Rising power increases electron density, which causes a higher concentration of active species to be present in the plasma [24]. The use of packaging material has a significant impact on how well a plasma sterilizing method works. Due to the minimal number of active species that passed through the packing, some of the spores that were inside became inactive. Instead, they combine again on the material’s surface or, more often, even interact with it.

The impact of CAP in packaging films include the changes in surface roughness, barrier properties (oxygen permeability and water permeability), contact angle, mechanical properties, thermal properties, anti-microbial properties, molecular properties, biodegradability etc. 

### 2.1. Surface Properties of Materials

The surface roughness properties of the packaging films will vary based on the power, treatment duration, feeding gas and energy of the plasma species along with the structure of the polymeric material. When DBD plasma, an RF-generated plasma, is utilized with a variety of gases, such as air, argon, O_2_, N_2_, etc., the etching effect is usually seen [25,26]. At atmospheric pressure, sputtering is minimal during plasma processing, and etching is primarily responsible for changes in surface morphology. Kostov et al. reported increased polymer degradation during the DBD processing is probably what causes the increased concentration of oxidized material on the surface. In this instance, in addition to being attacked by oxygen-related active species, the surface is also exposed to charged particles and powerful UV photons that have the potential to significantly break the polymer chain [27]. For N_2_ treated PEOT/PBR film, no appreciable variation in surface topography was seen, however the application of CAP substantially altered the surface topography by creating spherical entities on the film surface. The many active species that were present during the plasma discharge can be blamed for this variance in etching behaviour. Notably, an atomic oxygen species with outstanding etching properties was produced by air plasma with noticeably more oxygen molecules [28]. The type of feed gas can make significant changes in the etching process was experimented and reported by Fricke and co-workers. The primary action of argon plasma is sputtering caused by ion bombardment, whereas oxygen admixtures generate chemically active species that oxidize organic compounds and produce CO_2_ and H_2_O as a result. There may be a synergistic interaction between these two types of abrasion that boosts the etching power [29].

### 2.2. Barrier Properties of Materials

Food deterioration, inadequate storage, and decreased consumer compliance result from high oxygen and water permeability, which shortens the shelf life of packaged foods. The main purpose of barrier packaging is to protect packed goods from moisture/water, gas, and aroma throughout production, transportation, storage, and sale. Antimicrobial and antioxidant-rich active packaging materials help stop the spread of germs that can contaminate food [30]. Modern sensor technology is incorporated into smart/intelligent packaging materials to monitor food conditions and show their data. Near-field communication (NFC) and quick response (QR) barcode scanning are examples of smart packaging technologies that can gauge the freshness of the goods and give customers the most recent information about the products [31]. To functionalize cellulose and possibly hydrophilize its surface, an Ar/NH_3_ plasma is used, which leads to the creation of new polar groups. As a result, cellulose coatings that are extremely hydrophobic are created when exposed to Ar/SiH_4_ plasma. Ar/SiH_4_ or Ar/CH_4_ plasmas should be applied to the CNC film’s outer layer if you want to improve the oxygen barrier and prevent moisture through the deposition of thin hydrophobic films [32]. A surface coating thickness range that combines a very low oxygen permeation and insignificant residual stress effects was determined using gas barrier measurement data from Vasallo et al. on the plasma-induced deposition of SiO_x_ surface coating deposited from the HMDSO/O_2_ mixture on the PBS. Due to the extremely quick deposition time, the thin coating might enable industrialization from the perspective of process scalability [33]. The application of various gases enhanced the polar functional groups (such as peroxide, carboxylic acid, C–N, C=N, etc.) at the polymer surface and caused cross-linking, it had no effect on the Water or vapour permeability. This shows that it also depends on thermodynamic parameters such as vapour pressure, and concentration gradient across the film surface based on diffusion and the solubility parameters in addition to surface factors [34,35]. The decrease in oxygen permeability might be explained by increased cross-linking in polymer chains, which leads to a smaller free volume, which inhibits oxygen diffusion. According to several additional studies, plasma exposure causes the polymeric surface to undergo physiochemical modifications, such as the dissolution of the C–C and C–H bonds, which results in the production of free radicals. The formation of intermolecular and interchain bonds because of the interactivity of the produced free radicals with activated plasma species improves the oxygen barrier property [36,37].

### 2.3. Contact Angle for Wettability

One of the common ways to determine how wettable a surface or material is through contact angle measurements. The term “wetting” describes the spreading out of a liquid that has been deposited on a solid (or liquid) substrate or the capacity of liquids to form boundary surfaces with solid states [38]. The chemical and surface polarity, hydrophilicity etc. of the surface are all increased by cold plasma. Honarvar et al. investigated the effects of CAP treatment on polypropylene films coated with carboxymethyl cellulose having essential oil and discovered that the contact angle measurement decreased from 88.92° to 52.15° [1,39]. Ledari and coworkers also noted that after being exposed to cold plasma, the surface hydrophilicity of gelatine-based emulsion films greatly enhanced while the contact angle was significantly decreased [37]. The dielectric barrier plasma-treated starch film demonstrated an improvement in the contact angle values, defying the normal tendency where the application of LTP results in a decrease in contact angle. This might be caused by the –OH group being oxidized into C=O groups, which produces the new hydrogen bonds [40]. The potential for controlling the wettability of materials for packaging could open the door to new, specialized uses such coatings that are meant to release active compounds (like antimicrobials) slowly over time or other potential uses include water/moisture resistant coatings etc. were also investigated [41]. When cold plasma is applied to a film surface, the reactive species and free radicals amplify several polar groups, such as –COOH, –OH, and CO, which increases the film’s polarity, surface tension, and surface free energy. This process also makes the film surface more hydrophilic and wettable. Another likely explanation is the surface roughness brought on by the etching action, which facilitates the distribution of liquid onto the surface [42,43]. Overall, it can be said that exposure time, voltage, and plasma gas composition are all influences on the contact angle. The contact angle of a film’s surface is a crucial factor in determining whether it is suitable for packaging applications. A film’s surface’s coating, printing, absorbance, adhesion, and frictional qualities are influenced by its wettability [44,45].

### 2.4. Mechanical Properties of Materials

A series of crosslinking and breakdown events take place in the film structure during the cold plasma treatment of edible films, improves their mechanical properties [46]. In packaging applications, mechanical characteristics of the films, such as their tensile strength and elongation at break, are crucial factors in maintaining the quality and integrity of food while being handled, stored, and transported [39,47]. The polymer surface is typically activated to change or increase adhesion, printability, and integrity. According to Dong et al., extending the ACP treatment time enhanced the zein-based films’ tensile strength (TS), going from 10 MPa to 18.3 MPa, and their elongation at break (flexibility), going from 17 to 28.57% [43]. The bombardment of plasma on the film surface causes the cleavage of C–C and C–H bonds, which releases free radicals that form links with surface radicals or take part in chain reactions, improving the mechanical characteristics of film [36,43]. In addition to treatment duration, the gases utilized to produce plasma also have an effect by changing the mechanical characteristics of film. The tensile strength of the whey protein film was dramatically improved by applying air and argon plasma for 5 and 10 min, respectively. For air plasma treated film, extending the treatment duration up to 15 min greatly boosted the tensile strength; however, argon plasma treated film experienced a drop [36]. The rise in tensile strength may be due to the presence of carbonyl group cross-links formed following plasma treatment, which encourages hydrogen bonding and increases the structural integrity of the film surface [48,49]. Abdulkareem and colleagues reported that the Young’s modulus and stiffness have average values of 48.4 mN/m and 2.7 GPa, respectively for the untreated PLA film samples. Due to structural rearrangement brought on by etching, functionalization, ablation or crosslinking processes by plasma treatment, enhanced the nano-mechanical characteristics of plasma treated PLA films [50].

### 2.5. Thermal Properties of Materials

Several studies provide insight into how plasma treatment affects the thermal characteristics of films made of biopolymers. The two most crucial methods for comprehending the thermal characteristics and thermal stability are differential scanning calorimetry (DSC) and thermogravimetric analysis (TGA) [37]. The melting temperature (T_m_), glass transition (T_g_), denaturation (T_d_), and enthalpy (ΔH) of the samples are all determined by the DSC procedures, while the thermal stability of the samples can be verified by TGA [51]. Oh et al. reported the effects of LTP with several gases like oxygen, nitrogen, air, helium, and argon on edible films made from defatted soybean meal in their study. They noted that the addition of helium and argon raised the T_g_ (glass transition temperature) in comparison to the untreated film, which may have been caused by the radicals presence at the polymer surface, which caused a cross-linked network to develop and raised T_g_ [52]. When sodium caseinate film was subjected to DBD plasma treatment, Pankaj and Bueno-Ferrer [53] found that the plasma-treated film’s T_g_ was lower than that of the untreated film. Chemical etching caused by the dissolution of chemical bonds, chain scission or chemical degradation can be attributed for the drop in T_g_ [53]. Cold plasma treatment can induce positive or negative thermal properties.

### 2.6. Surface Chemistry Properties

An analysis of the biopolymer film at the molecular level sheds light on the molecular interactions among multiple elements that make up the film network. Understanding molecular interactions is crucial since they affect the film’s mechanical, thermal, and physical characteristics. Fourier transform infrared (FTIR) spectroscopy is one of the most used techniques for examining structural alterations in the biopolymer film [48]. Protein N–H and O–H stretching is what causes the recognizable peak in the 3700–3000 cm^−1^ range. Higher polar groups on the material’s surface caused a shift and a minor rise in peak intensity at 3300 cm^−1^ after film was treated with atmospheric cold plasma [43]. According to studies, LTP treatment of starch-based films introduces oxygen containing functional groups into the film, increasing hydrogen bonding and crosslinking. With the aid of air plasma, Arolkar et al. investigated how corn starch/PCL films could be structurally modified. They found that the intensity of peaks at about 3600–3000, 1265, and 1016 cm^−1^ increased, indicating an increase in oxygenated functional groups like O-H. The interaction between oxygen-rich molecules in the plasma and the film may be the cause of this rise in peak intensity [54].

### 2.7. Anti-Microbial Properties of Materials

Innovative approaches to the rise in food-borne illnesses and alterations in eating patterns include active packaging and antimicrobial and anti-fungal surfaces. By decontaminating packing surfaces, the cold plasma method enhances the safety of food products in addition to changing the surface qualities of biopolymer-based films. When exposed to LTP, the active packaging sheets that are filled with bioactive substances like peptides, functional ingredients or essential oils exhibit increased antibacterial activity [55].

**Figure 2:**
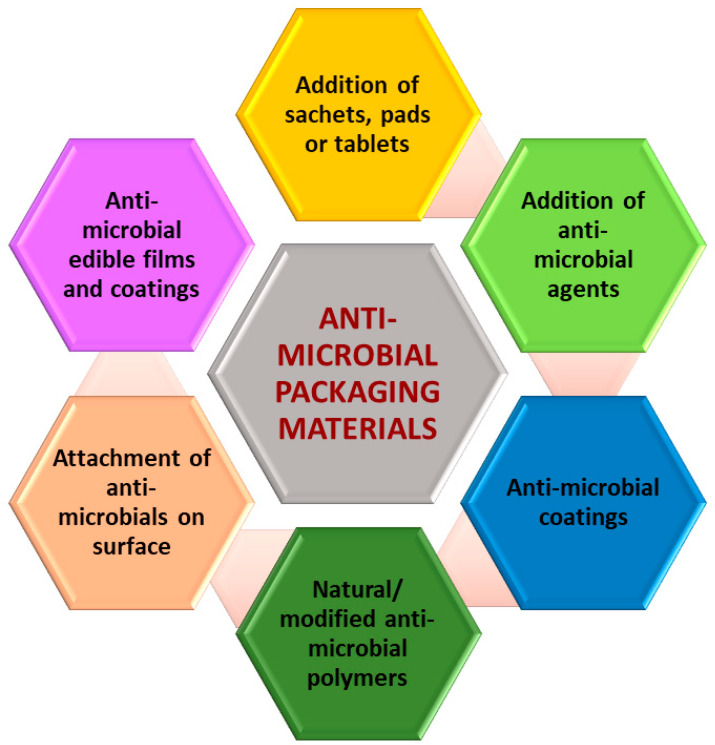
Different methods for the fabrication of anti- microbial active surfaces.

Different methods are already discovered for making anti-microbial active material surfaces. These methods are classified based on the mode of anti-microbial incorporation (Figure 2). By adding active ingredients to the container using a sachet, pad, or tablet and allowing processes like evaporation and absorption to prevent the microbial development, the environment within the packaging can be modified. The potential of sachet leakage and the possibility of sachet ingestion by accident are two major drawbacks of such sachets, which are enclosed loosely or affixed to the interior of a packaging [56].

To obtain the proper controlled release of anti- microbial agents to the food surface, antimicrobials can be added to multilayer films with control, matrix, and barrier layers. The barrier layer stops the agent’s migration toward the package’s exterior while the matrix layer holds the agent, and the inner layer regulates the rate of diffusion of the active component [57]. Numerous antimicrobials can be added directly to packaging materials, especially films. Applying the antimicrobial chemicals as a coating is also an option to incorporating antimicrobial compounds during extrusion. The benefit of doing this is that the specific antimicrobial ingredient may be added in a controlled way without being exposed to high temperatures or shearing pressures [58]. Some bioactive surfaces have been created by altering the surface of polymers, in contrast to naturally antibacterial polymers. Due to issues with the environment, the need for improved storage methods, and chances to open new markets for underutilized agricultural products with film-forming abilities etc. paved the way for increasing interest in edible coatings too [59].

It has been found that plasma treatment makes it easier for functional components to be coated on the polymer layer, increasing the antibacterial effectiveness. For instance, Wong and co-workers created a polyethylene film coated with gallic acid using a plasma treatment (30 W for 60 s). At a concentration above 1.0%, the PE/GA film inhibited the growth of *E. coli* and *S. aureus* bacteria by 0.5–1.1 logs [60]. When exposed to cold plasma, the packaging films that are filled with bioactive exhibit increased antibacterial activity. A key factor affecting the anti- microbial activity of the films can be the release rate and diffusion rate of antimicrobial agents from the cold plasma-treated polymer matrix [61].

### 2.8. Biodegradability of Materials

The capacity of packaging sheets to biodegrade and decompose through the enzyme-mediated processes of living microorganisms in environmental settings sets them apart from synthetic packaging. According to Arolkar et al., investigation into the breakdown of corn starch/PCL films after soil burial, the degree of bio degradation was estimated in terms of a change in TS and EAB. The rate of bio degradation was found to be higher for air plasma functionalized films than control films, and it grew as the air plasma treatment period increase [54]. Another study reported the degradation of PLA sachets for 0 to 35 days and found that, after 28 days, the extent of bio degradation of PLA sachets functionalized with plasma was greater than that of controlled sachets. The treated PLA sachets entirely decomposed after 35 days, however the untreated sachets remained intact [42]. The surface functionalization-induced alterations are time-dependent, which is a significant disadvantage of plasma treatments. This “aging” or “hydrophobic recovery” process is ascribed to structural rearrangement that bury chemical compounds placed at the surface as well as migration of polar function groups into the polymer bulk [62].

## 3. Plasma Technology in Food Packaging

Apt packaging is essential for maintaining and extending the shelf life of fresh produce, and the inclination to utilise non-thermal innovative technologies to extend the shelf life of fruits and vegetables has developed more recently than traditional techniques as a result of the need for environmental protection and energy conservation. Researchers in the packaging business believe it is crucial to use packaging materials to reduce the rate of respiration, the production of ethylene, the rate of deterioration, and the microbiological activity of fresh fruit and vegetables [63,64,65]. These technologies not only preserve the product’s aesthetic qualities but also result in less modifications to its qualitative attributes [66]. The information below is a thorough compilation of information drawn from recent research on the impact of plasma treatment on various systems in the food and pharmaceutical packaging industries (Table 2). It can be understood that the plasma treated samples, irrespective of the treatment conditions, always exhibited superior properties when compared to their pristine counterparts. The detailed understanding of the tabulated studies are also explained in latter sections.

As evidenced from the table above, different types of plasma treatments have been adopted by different researchers, and the treatment effects on the resultant composite and substrate properties seem to correlate or vary depending on the type of plasma treatment, type of raw materials, and intended end use applications. As shown in the table, majority of the studies were carried out using dielectric barrier discharge (DBD) cold plasma treatment, where researchers like Rashvand et al. [65], Goiana et al. [72], Wu et al. [77] and Chen et al. [47,79] reported improvements in tensile strength, elasticity and barrier properties like water vapour and oxygen transmission rates. Enhanced stability and shelf life of packaging films and substrates were observed by Glicerina et al. [67] and Akhavan-Mahdavi et al. [68], and modified surface properties of composite materials were reported by Goiana et al. [72] and Chen et al. [79], where they noted changes in surface morphology and hydrophobicity depending on the type of film and plasma treatment frequency, with improved wettability, roughness, and surface free energy after coating or plasma treatment. Additionally, changes in film structure were observed by Wu et al. [77] and Chen et al. [47], while observing rearrangement of protein chains, slight changes in crystal structure, and improved packing density within the film structure. Chen et al. [79] described better-ordered porous structure and enhanced compatibility between zein and coating materials, while Chen et al. [47] reported ordering of zein molecule secondary structure after plasma treatment. Cold plasma treatment, in general, were carried out by a few researchers including Yudhistira et al. [73], Cui et al. [76] and Lin et al. [81]. The major effects on composite properties reported by them include improved coating adhesion, controlled release of active agents and surface modification for enhanced antimicrobial activity. It is interesting to note that unlike the DBD cold plasma treatment, the enhancement of mechanical and barrier properties have not been significantly reported in general cold plasma treatment.

The key findings from two reported studies employing DBD atmospheric air cold plasma (DBD-ACP) include surface modifications, interfacial interactions, and stability and delamination resistance. Quite different from other reports, the use of DBD-ACP exhibited increased hydrophilicity, suggesting improved water absorption and wetting properties. Both Heidemann et al. [80] and Li et al. [70] observed enhanced adhesion and interaction, whether it be between film layers, or between the film and food materials, thereby leading to an improvement in barrier properties and film functionalities. Nevertheless, helium cold plasma treatment carried out by da Fonseca de Albuquerque et al. [71] did not significantly influence the water vapour permeation, but created an improved physical barrier to water. Moreover, even though this treatment technique increased the surface roughness, it was not significant enough in increasing the water contact angle of the modified surface. Surface dielectric barrier discharge (SDBD) plasma from Plasma Assisted Sanitation System (PASS) is another less common technique reported by Capelli et al. [74], predominantly used for food packaging decontamination from SARS-CoV-2 RNA. In this study, they observed that the plasma treatment decontaminated virus, without significantly affecting the properties of packaging and food substrate. Yet another plasma treatment method termed carbon tetrafluoride (CF_4_) reactive-ion etching (RIE) was reported by Kim et al. [78], where nanopillars are introduced to the film surface, with potential applications in developing antibacterial overcoating. Such films were observed to exhibit improved optical and antibacterial properties.

Overall, these reports indicate that various coating technologies can effectively enhance the functional characteristics of food packaging films, resulting in augmented product protection, prolonged shelf lives, and possibly improved sustainability. However, it’s important to note that explicit effects of each coating technology can depend on the type of film material, coating composition and treatment parameters.

The prospects of using plasma treated sodium alginate (SA) films for food packaging applications was studied by Sharmin and colleagues [75], where, unlike the traditional methods of plasma treatments, this study dealt with the use of plasma activated water (PAW). The main focus of the researchers remained on understanding the effect of citric acid (CA) and PAW on the mechanical, rheological and barrier properties of SA films (Also given in Table 2). The tensile strength and elongation at break values of the resultant films were seen to increase by 43 and 66% when PAW was used to prepare SA solutions, while a reduction in water vapour transmission rate (WVTR) up to 44% was obtained for 1% CA incorporated SA samples, all indicating the development of better films when compared to neat SA films.

**Figure 3:**
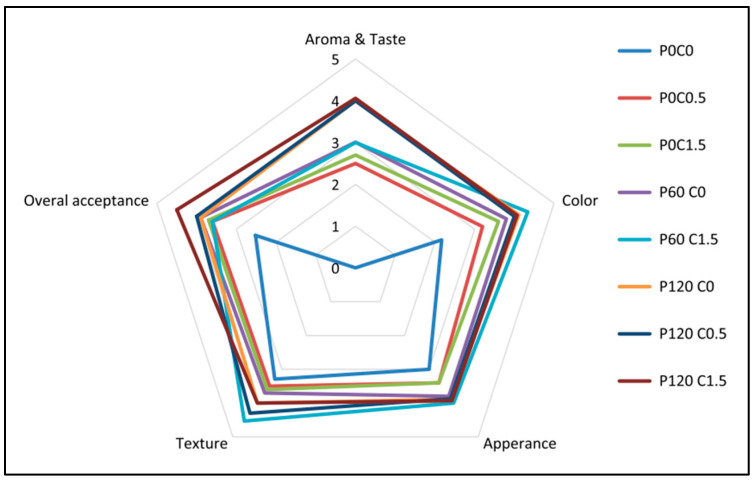
Sensory evaluation results of pistachio treated with chitosan and plasma after 180 days of storage. Reproduced from Akhavan-Mahdavi, S.; Mirzazadeh, M.; Alam, Z.; Solaimanimehr, S. The Effect of Chitosan Coating Combined with Cold Plasma on the Quality and Safety of Pistachio during Storage. Food Sci. Nutr. 2023, 11, 4296 [68], under the conditions of the Creative Commons Attribution (CC BY) license.

Effect of different chitosan concentrations and plasma treatment durations on fresh pistachios was studied by Akhavan-Mahdavi and co-workers [68], and the radar chart depicted below provides an in-depth understanding on the sensory analysis results after 180 days of storage of pistachios after the treatments (Figure 3). In their study, the influence of varying amounts of chitosan (0.5 and 1.5% *w*/*v*) and cold plasma treatment duration (60 and 120 s) were observed and reported for 180 days of pistachio storage. The resultant effects on the pistachio shelf life were estimated by measuring various factors such as hardness, colour components, total mould and yeast, moisture and aflatoxin content, peroxide values, and sensory evaluations. From the reported results, it can be seen that the treatment with 1.5% chitosan and 120 s of cold plasma treatment produced highest conservation of pistachio hardness and colour indices. It has also been reported that among all the examined parameters, the plasma treated sample P_120_ C_1.5_ and the control sample exhibited the highest and lowest scores respectively.

While studying the decontamination of food packages from SARS-CoV-2 RNA with a cold plasma-assisted system, Capelli and team observed that plasma treatment decontaminated the virus, without significantly affecting the properties of either the packaging or the food substrate [74]. As reported for K5 and K10 samples, the viral RNA reduction driven merely by air exposure for both the packaging materials tested was very minimal. Both packing materials underwent a considerable but modest reduction in detectable RNA after 5 min of CAP treatment of around 10 and 16% for PP and PET, respectively. The RNA molecules, whose quantity was below the detection thresholds for each target sequence discovered by this PCR approach, were fully destroyed by CAP treatment for 10 min. Both PET and PP were used to study how O_2_, and CO_2_ changed in the headspaces of the packages as they were being stored. As predicted, the respiration of apple tissues resulted in CO_2_ building up and O_2_ being used up. Due to the greater thickness and reduced permeability of PET, the O_2_ loss was accelerated for apples in packaging. Also, O_2_ had been completely absorbed in just three days. However, for both packing materials, there was no discernible variation in CO_2_ and O_2_ concentration between treated (P5, P10) and control samples (C) [74]. The apple samples were also measured for titrable acidity (TA) and firmness values (N) during storage. TA showed an increase during storage in all apple samples packaged in PE, which was attributed to the generation of carbonic acid from CO_2_, which is present in higher concentration in PET packages than in PP ones. Although the exposure to CAP did not significantly affect the samples in either case, this difference was correlated with the type of components present.

**Figure 4:**
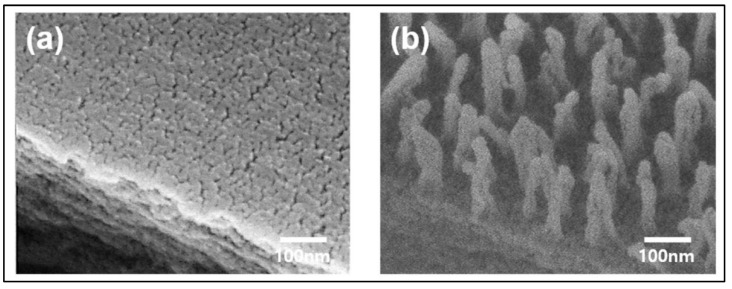
FE-SEM 45°-tilted images of (**a**) bare PET and (**b**) CF_4_-RIE PET. Reproduced from [78] Kim, J.H.; Mun, C.; Ma, J.; Park, S.G.; Lee, S.; Kim, C.S. Simple Fabrication of Transparent, Colorless, and Self-Disinfecting Polyethylene Terephthalate Film via Cold Plasma Treatment. Nanomaterials 2020, 10, 949, under the conditions of the Creative Commons Attribution (CC BY) license.

The morphological changes induced by plasma treatment on PET surface has been reported by Kim et al., and the images are given in Figure 4 [78]. It was found that the CF_4_-RIE PET had a regular array structure made up of spherical capped nanopillars with 30 nm diameters, 237 nm heights, and 75 nm pitches (the space between two pillars), as opposed to the flat surface of neat PET. The *Progomphus obscurus* (sand dragon) wing, which exhibits a nanopillar array structure composed of high-aspect-ratio spherically capped nanopillars with an average diameter of 50 nm and an average height of 241 nm, served as the source of inspiration for the CF_4_-RIE’s nano surface structure. The optical improvement observed by the researchers were also attributed to these nanopillar array structure, where the rough surfaces tend to reduce transmittances by increasing the absorption capacity for incident light due to light scattering.

While studying the effect of cold plasma treatment on thyme essential oil (TO)/silk fibroin (SF) nanofibers against *Salmonella Typhimurium* in poultry meat, Lin and co-workers [81] conducted sensory evaluation tests were on chicken and duck meat. According to them, when compared to the control group, chicken and duck meat samples wrapped in plasma-TO/SF nanofibers membranes demonstrated an improvement in flavor and general appeal. The researchers also deduced that the sensory evaluation scores of poultry meat with plasma-TO/SF nanofibers were higher than the control group since the test group received a higher score after the plasma treatment, indicating that plasma-TO/SF nanofibers membrane could improve food quality without losing the good flavor.

As part of the U.S. National Science Foundation supported program on Future Technologies Enabled by Plasma Process (FTPP), at the University of Alabama at Birmingham (UAB), we focused on the developmental studies, a step further, to have smart sensor-integrated packaging against food pathogens or toxins via inkjet printing onto plasma treated films [2,82]. Moreover, our group recently published an invited mini review on mitigation strategies in engineered healthcare materials towards antimicrobial applications and another on non-thermal plasma processing for nanostructured biomaterial [83,84]. Plasma research programs for materials and biomatter for application in agriculture, medical materials [85,86,87,88,89,90,91,92,93,94], food packing, and other plasma-technologies for automobile and aerospace applications will establish Alabama State as a Southeastern regional hub for plasma science expertise and create thousands of high-paying technical careers in the state and region.

## 4. Conclusions

The production of the bulk of plastic packaging involves fossil fuels, which has a negative influence on the environment. Plastic packaging developed from bio-based materials is a potential improvement in sustainability. One of the most encouraging and alluring solutions for future sustainable packaging, in particular, is expected to be biodegradable bio-based materials with improved barrier properties, thermal and mechanical properties, anti-microbial properties etc. Given that, it is a straightforward inline process with a simple instrumental setup and no waste generation, applying robust cold plasma (or LTP) treatment to biopolymer-based films is an economically advantageous, green and easy way to improve their packaging qualities. The efficacy of in-package plasma therapy can be impacted by the kind of plasma gas, the composition of the biopolymer structure, and the processing parameters (voltage and treatment time). Various studies reported that the plasma treatments will improve the surface properties of packaging materials without affecting the bulk properties of the packaging material in a sustainable way. Overall, it can be said that using low temperature plasma to overcome the drawbacks of biopolymer-based packaging materials for use in food packaging is an efficient technique. The studies regarding the aging stability of surface chemistry and functional groups of various plasma processed materials, plasma polymerized materials, plasma-grafted materials are still an ongoing debate. Collectively, these reports contribute to a comprehensive understanding of the multifaceted improvements in food packaging materials, ranging from mechanical and barrier enhancements to surface modifications and food safety considerations. Future research could focus on optimizing coating processes for specific food applications and developing novel coating materials with tailored functionalities. Maintaining the long-term stability and controlled release profiles of active agents within the films is crucial for their effectiveness. The long-term stability and potential migration of plasma-induced modifications from the film surface need further investigation. In the end, food producers and consumers stand to gain from these quality enhancements as they can increase the attractiveness and marketability of food items.

## Figures and Tables

**Table 1:** Important plasma sources used in food packaging applications.

CAP Generation Methods	Schematic Diagram	Features	Reference
Dielectric Barrier Discharge (DBD) Plasma	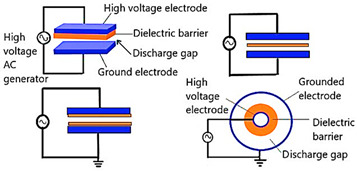	Planar or cylindrical consist of an insulating electrode and a grounded electrode. The DBDs are scalable, efficient, and have short processing times [10,11]. They also consume less energy. The high ignition voltage and the narrow discharge gap height, which are both connected with plasma homogeneity, are the main downsides, though.	Reprinted from [10] under under the terms of the Creative Commons CC-BY license and copyright permission from Elsevier
Corona Discharge Plasma	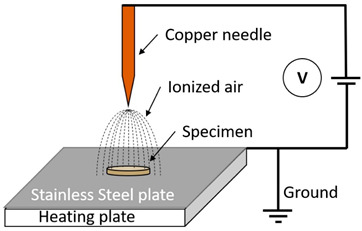	A runnel of charged particles, including ions and electrons, is called a corona, and it is accelerated by an electric field. It is created when a gaseous space gap, such as one containing air or another gas, is exposed to a voltage high enough to cause a series of high-velocity particle collisions with neutral molecules, leading to the creation of more ions. One of the noticeable advantages of corona discharge is the energy consumption to breakdown the gas is very low. Also, since the supply voltage is very less than DBD, it is very safe to use. But compared to the DBD plasma, overall, the processing effect of corona discharge is very weak, and the concentration of charge can cause electron damage soon [12,13].	Reprinted from [12], under under the terms of the Creative Commons CC-BY license
Atmospheric Pressure Plasma (APP)	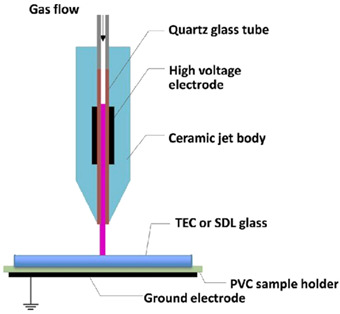	A neutral gas under an electrical field is the primary source to produce the APP. A gas is excited using direct current or alternating current at frequencies varying from low to several GHz while it is under atmospheric pressure. For plasma creation, the chosen plasma gas is often accomplished between two plates. Continuous treatments can be done using APP. So, this process is very cost effective and strong. The initial setup for the process is not economical [14,15].	Reprinted from [14] under under the terms of the Creative Commons CC-BY license
Gliding Arc Discharge Plasma	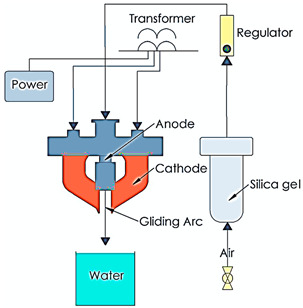	An atmospheric pressure arc discharge known as a “gliding arc” can produce very high levels of electron density, current, and power as well as a relatively low temperature and an enhanced electric field, which are common of cold atmospheric plasmas. Improved damping ratio is one of the important advantages of gliding arc discharge plasma [16,17]. In some cases, severe overheating can be a potential drawback of this method. This can cause heating of the medium	Reprinted from [16] under a Creative Commons Attribution-NonCommercial 3.0 Unported Licence.
Radio Frequency (RF) discharge Plasma	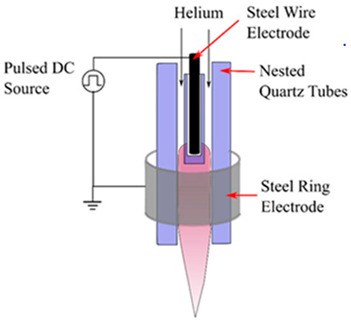	A needle electrode’s tip generates plasma, which spreads outside the ceramic nozzle to a grounded ring electrode attached to the two RF voltage electrodes (of which a frequency of 13.56 MHz is typically utilized). The spatial spread of RF plasma is not constrained by electrodes. It will prevent metallic vapours from contaminating the plasma. RF plasmas are widely recognized for producing plasmas with high electron densities [18].	Reprinted from [19]

**Table 2:** Detailed description from recent reports on the effect of plasma treatment on different systems in the field of food and pharmaceutical packaging.

No.	Plasma Treatment Conditions	Matrix and Fillers; Composite Type	Applications	Effect of Plasma Treatment on Properties	References
1.	Dielectric barrier discharge (DBD) cold plasma, for 5, 10 and 15 min. Maximum transmission power: 50 W; Voltage: 15 kV; Current: 10 mA; Frequency: 50 kHz; Power source: DC pulse type with pulse width modulation (PWM)	Chitosan + cellulose nanoparticles; Films	Packaging of strawberry	For films: Improved mechanical properties (TS & EAB), water vapour permeability, oxygen transmission rate, moisture content and water contact angle. For substrates: Enhanced mechanical properties (firmness and Young’s modulus), chemical attributes (pH, soluble solid content and total ascorbic acid), physical characteristics (weight loss and colour features), microbial activities (bacteria, yeast and mould)	[65]
2.	Open-air DBD cold plasma. Peak voltage: 20 kV; Frequency: 20 kHz	Polylactic acid multilayer films	Active packaging of sunflower oil and “pesto” sauce; Biodegradable multilayer active packaging, to extend food products shelf-life and/or maintain high quality levels of oily foods during storage.	Immobilization of oxygen scavenger agent (ascorbic acid); Decreased oxidation kinetics; Better and more stable quality characteristics in terms of colorimetric, microbiological and textural parameters	[67]
3.	DBD cold plasma, for 60 and 120 s. Gas source: Air; Argon gas type, oxygen gas pressure of 0.4 millibars equivalent to 0.3 Torr and power of 89 watts equivalent to radiometric waves	Chitosan solution	Preservation of quality and safety (shelf life) of pistachios during storage	Significant reduction in the amount of aflatoxin, mold and yeast after 120 days; Physicochemical characteristics of pistachios did not change significantly; No adverse effect on the sensory characteristics of pistachios	[68]
4.	Atmospheric air cold plasma treatment for 5, 10 and 15 min in the excitation mode. Input voltage: 6.2 kV; Power level: 60 kW; Pulse frequency: 10 kHz	Wild almond protein isolate (WAPI) + Persian gum (PG); Films	Edible films in food packaging	Progressively improved mechanical properties (increased thickness, TS and EAB); No significant effect on WVP and solubility; Surface roughness directly proportional to plasma treatment time, but surface remained integrated; Best results obtained for films with 10 min treatment; Properties tend to deteriorate after 15 min treatment	[69]
5.	Dielectric Barrier Discharge Atmospheric Cold Plasma (DBD–ACP); Fixed exposure time (3 min) with varying voltages of 10, 20, 30, 40, and 50 kV; Fixed voltage (30 kV) with varying exposure times (1, 2, 3, 4, 5 min)	Soy protein films	Edible packaging and food preservation	Increased water interactive properties and thermostability; Decreased surface roughness; Effects of different ACP treatment times too	[70]
6.	Cold plasma based on helium. Glow discharge reactor at 13.56 MHz. Chamber vacuum: <8 Pa. Treatment with He: Self-bias voltage −100 V; Treatment time: 10 min. Treatment with HMDSO: Self-bias voltage −60 V; Treatment time: 20 min	Hexamethyldisilox-ane (HMDSO) treated extruded corn starch films	Barrier films for food packaging and pharmaceutical products	More homogeneous coating and smaller granules; Increased hydrophobicity, but roughness created by helium plasma was not effective in increasing the water contact angle of the modified surface; No much effect on water vapour permeation; Significant reduction in absorbed water content, mostly due to the formation of a barrier to water absorption of around 80%; Physical barrier to water, while allowing permeation to water vapour	[71]
7.	DBD cold plasma treatment. Voltage: 20 kV; Excitation frequencies: 50, 400 and 900 Hz; Treatment time: 5 min	Starch, gelatin and bacterial cellulose films	Sustainable and biodegradable alternatives for plastic packaging	Improved hydrophobicity, surface morphology, tensile strength, and elasticity module; Reduced water solubility; Pronounced changes for starch films at low excitation frequency (50 Hz) of plasma, and for gelatin and bacterial cellulose films at high excitation frequency (900 Hz)	[72]
8.	Cold plasma treatment. Vacuum plasma reactor. Frequency: 13.56 MHz; Pressure: 0.0643 Torr; Power: 30 W; Treatment time: 60 s	LDPE + *Myristica fragrans* Essential Oil (MFEO); Films	Active food packaging material	Cold plasma treatment improved the properties of LDPE films by facilitating MFEO coating	[73]
9.	Surface dielectric barrier discharge (SDBD) plasma from Plasma Assisted Sanitation System (PASS) for 5 and 10 min. Gas: Environmental air; Relative humidity: 20–40%; Voltage: 1–20 kV; Frequency: 1–20 kHz; Tunable duty cycle: 1–100%. Imposed voltage: 6 kV; Frequency: 5 kHz; Fixed duty cycle: 100%	Polyethylene terephthalate (PET) trays (350 microns thick) and polypropylene (PP) film (69 microns thick)	Newly developed plasma sanitation system for food packaging decontamination from SARS-CoV-2 RNA	Plasma treatment decontaminated virus, without significantly affecting the properties of packaging and food substrate; 5-min treatment reduced detected RNA for both surfaces, but to different extents. Indicated that interaction between reactive species and viral genetic material is affected by the matrix; 10-min treatment completely degraded RNA molecules from both surfaces	[74]
10.	Plasma activated water (PAW) produced using surface barrier discharge (SBD) sourced high voltage cold plasma (CP). Sinusoidal signal frequency: 18 kHz; Atmospheric pressure; Plasma-inducing gas: Room air	Sodium alginate films	Food packaging	Increased TS, tensile modulus, EAB, LVE region and storage modulus; No intersection between G′ & G″; Showed shear thinning properties or non-Newtonian behaviour; decreased WVTR	[75]
11.	Cold plasma treatment. Treatment time: 30 s; Power: 350 W; Nitrogen flow rate: 100 standard cubic centimeters/min (sccm)	Momordica charantia polysaccharide (MCP) nanofibre + Phlorotannin (PT); Electrospun nanofibre membranes	Active food packaging	Increased release efficiency of PT, resulting in an increase in antibacterial and anti-oxidant activities, without the alteration of chemical structure	[76]
12.	DBD cold plasma. Voltage changed group adjusted at a changed treatment of 0, 30, 40, 50, 60 and 70 V under the duration of 60 s. Time changed group subjected to a sustaining time of 0, 15, 30, 45, 60, 90 and 120 s under the voltage of 50 V; Current: 2 ± 0.2 A	Casein edible films	Packaging material	Crystalloid migration and casein aggregation (via SEM) leading to reinforcement of structure stability; Slight change in crystal structure (via XRD); Stable state of molecular structure (via FTIR); Remarkable improvement in packing characters (including mechanical and barrier properties); Slight modifications of colour and transparency; Rearrangement in order of protein chains	[77]
13.	Carbon tetrafluoride (CF_4_) reactive-ion etching (RIE) using 13.56 MHz radio-frequency plasma equipment. Flow rate: 3 sccm; Working pressure: 3.0 × 10^−2^ Torr; Treatment time: 4 min; Power: 100 W	Transparent, colourless and self-disinfecting polyethylene terephthalate (PET) film that mimics the surface structure of *Progomphus obscurus* (sand dragon) wing, physically killing the attached bacteria	Antibacterial overcoating with good optical properties for contactable surfaces in private and public interior spaces and packaging applications	Introduction of nanopillars; Improved optical properties (transparency and colourlessness); Notable enhancement in antibacterial activity against *S. aureus* and *E. coli* by activating or strengthening physical biocidal action	[78]
14.	Cold plasma (CP) generated by dielectric barrier discharges (DBD) plasma reactor. Voltage: 60 V; Current: 1.5 A. Short-term treatment time: 60 s; Long-term treatment time: 120 s	CP pre-treated zein films + Porous PLA layer coating by breath figure self-assembly	Biodegradable packaging	Better-ordered porous structure after coating with PLA; Induced compatibility between zein and PLA molecules, by changing the protein conformation and by enhancing the intermolecular hydrogen bonding interactions; Significant improvement in surface hydrophobicity, fracture resistance, water vapor barrier, and thermal stability; Improved UV barrier and excellent biodegradability; Potential to enhance adhesion and improve functionalities of porous coating on other biopolymer materials	[79]
15.	DBD atmospheric air cold plasma (at ambient temperature and atmospheric pressure). Plasma discharge frequency: 50 Hz; Voltage: 31 kV; Treatment time: 1, 5, 10, 15 and 20 min	Polycaprolactone (PCL) or poly(lactic acid) (PLA) and cassava starch multilayers	Multilayer packaging materials	Increased hydrophilicity and surface roughness; Improved adhesion between layers, zeta potential, delamination resistance, etc.	[80]
16.	Cold plasma treatment. Power: 400 W; Treatment time: 4 min; Nitrogen flow rate: 100 sccm	Silk fibroin nanofibers + Cold plasma treated thyme essential oil (TO) composite films, post-treated with cold plasma	Effective antimicrobial packaging to increase shelf life of foods	Increased antibacterial activity by increasing TO release amount, due to surface modification, but without affecting chemical composition of the films; Decreased number of Salmonella Typhimurium in chicken and duck meat	[81]
17.	DBD-50 cold plasma reactor. Power: 100 W; Treatment time: 30, 60, 90, 120 and 150 s	Zein + Chitosan films	Food and pharmaceutical packaging materials	Improved wettability, TS, EAB, water vapour barrier and thermal stability; Secondary structure of zein molecules became ordered; Rougher surface morphology, increased surface free energy and enhanced hydrogen bond interactions between zein and chitosan after plasma treatment (optimum range: 60–90 s)	[47]
